# Low CT attenuation and high fatty infiltration rate of psoas are risk factors for incisional hernias after appendicectomy: a cross-sectional single-center study from China

**DOI:** 10.1186/s12891-021-04333-2

**Published:** 2021-06-30

**Authors:** Xuechao Du, Pengtao Sun, Yuchang Yan, Xiang Gong, Yufei Lian, Zhenyu Pan

**Affiliations:** 1 Department of Radiology, Beijing Chaoyang Hospital, Capital Medical University, 5 Jingyuan Road, Shijingshan District, Beijing, 100043 China; 2 Department of Radiology, Beijing Shijitan Hospital, Capital Medical University, Beijing, 100038 China

**Keywords:** Incisional hernias, Psoas, Computed tomography, Intramuscular adipose, And sarcopenia

## Abstract

**Background:**

Decreased computed tomography (CT) attenuation of muscle is independently associated with muscle weakness. The CT attenuation of the abdominal wall muscles may correlate with that of the psoas in patients without ventral hernias. This means that the CT attenuation of the psoas may be related to the occurrence of incisional hernias (IH). CT-determined sarcopenia was deemed inefficient in predicting the development of IH, while limited attention has been paid to the association between muscle fatty infiltration and incidences of IH. In this study, we aim to investigate whether the psoas’ CT measurement parameters, including the average CT attenuation, fatty infiltration rate and psoas muscle index, are associated with IH.

**Methods:**

In this study, adult patients who had undergone an appendicectomy in the past and had then, for any reason, been hospitalised in our hospital from January 2018 to December 2019 were enrolled. The patients were classified into an IH group and a non-IH group. Their psoas’ CT attenuation, fatty infiltration rate (FIR) and psoas muscle index (PMI) were measured or calculated. Sarcopenia was defined according to their PMI. Differences between the two groups’ indices were then compared. A logistic regression model was applied to assess the effects of psoas’ CT measurement parameters on the occurrence of IH.

**Results:**

One hundred twenty patients were included in this study. The psoas’ CT attenuation (*p* = 0.031) and PMI (*p* = 0.042) in the IH group were significantly lower than those in the non-IH group, and FIR in the IH group was significantly higher than in the non-IH group (*p* < 0.001). The patients’ psoas’ CT attenuation, FIR, PMI, age, gender and whether they had a history of smoking, were all significant factors in the univariate logistic regression analysis. After adjusting for confounding factors, a multivariate logistic regression analysis demonstrated that the psoas’ CT attenuation was an independent protective factor (*p* = 0.042), and FIR was an independent risk factor (*p* = 0.018), while neither PMI (*p* = 0.118) nor sarcopenia (*p* = 0.663) showed a significant effect on the incidence of IH.

**Conclusions:**

When an appendectomy has been performed, a decreased CT attenuation and increased FIR of the psoas can be considered risk factors for IH.

## Background

Sarcopenia is considered a patient-specific imaging biomarker able to predict clinical outcomes. Several imaging modalities can be used to assess muscle mass and to diagnose sarcopenia, including dual-energy X-ray absorptiometry (disadvantage: creates inhomogeneous results among different densitometer brands), computed tomography (CT, disadvantage: high radiation exposure), magnetic resonance imaging (MRI, disadvantages: high equipment costs and low availability), and ultrasound (disadvantages: low reproducibility and low accuracy) [1]. CT, which is routinely performed for evaluating abdominal diseases, is optimal for opportunistically assessing sarcopenia without the need of additional examinations or radiation exposure. Besides diagnosing sarcopenia by measuring muscle size in specific districts, CT can also quantitatively measure muscle attenuation and fatty infiltration. All these CT measurement parameters can reflect muscle degeneration.

Muscle weakness in the abdominal wall is one of the main factors that causes ventral incisional hernias (IH). A decreased CT attenuation of muscle is independently associated with muscle weakness [2]. Therefore, the decrease of CT attenuation in the abdominal wall muscles may be a risk factor for IH. When a ventral hernia develops, the abdominal wall is passively unloaded, resulting in atrophic changes in the unloaded skeletal muscles [3]. Since the abdominal wall muscles have a different status before and after the occurrence of a ventral hernia, the CT attenuation in the abdominal wall muscles of patients with IH cannot accurately reflect the status of abdominal wall muscles before the occurrence of a hernia. The CT attenuation of the abdominal wall muscles may correlate with that of the psoas in patients that do not have a ventral hernia [4]. Therefore, we hypothesise that the CT attenuation of the psoas is related to the occurrence of IH. CT-determined sarcopenia has been shown to be an independent and unfavourable predicting factor for various abdominal diseases [5–7]. However, a few studies have concluded that CT-determined sarcopenia is not sufficient to predict the development of IH [8]. Another indicator, and one that very limited attention has been paid to, is the association between muscle fatty infiltration (another indicator of muscle degeneration) and IH incidences. In this study therefore, we aim to investigate if the psoas’ CT measurement parameters, including average CT attenuation, fatty infiltration rate (FIR) and psoas muscle index (PMI), are associated with IH. This is explored by measuring the psoas’ parameters on the CT images from both IH and non-IH patients that have undergone an appendectomy procedure due to appendicitis in the past.

## Methods

Adult patients (> 18 years old) who have had an appendicectomy due to appendicitis and were hospitalised in our hospital from January 2018 to December 2019, were consecutively enrolled in this study. The inclusion criterion was: patients had undergone an abdominal CT examination during their hospitalisation. The exclusion criteria were: (a) various factors that could affect muscle mass, including malignant tumours, taking medicines such as glucocorticoids or thyroxine, endocrine diseases, chronic exercise limitations, organ failure and uncontrolled cardiopulmonary diseases; (b) having a lumbosacral transitional anatomy; and (c) having ventral hernias in areas other than appendicectomy incisions. The patients were classified into an IH group (clinically or surgically confirmed incisional hernias at appendicectomy incisions) and a non-IH group. Data on age, gender, height, weight, a history of smoking, hypertension and diabetes were recorded. Body mass index (BMI, kg/m^2^) was calculated by dividing their weight in kilograms by the square of their height in metres. Our hospital’s Research Ethics Review Committee reviewed and approved this study. All methods in this study were carried out in accordance with the Helsinki guidelines and declaration.

### CT scan and image measurements

The CT scans were performed using gemstone CT spectroscopy (Discovery CT 750 HD, GE Healthcare). Imaging protocols included: 120kVp, tube current modulation, pitch 0.984, slice thickness 5 mm, slice spacing 5 mm. Reconstructed images with a 0.625 mm slice thickness were obtained for further analysis. CT measurements were independently carried out by two experienced radiologists. At the GE AW 4.6 workstation, bilateral psoas muscles were measured separately at the level of the third lumbar vertebra (L3), upon which both the transverse processes were visible, as previously described [9]. By manually outlining both the left and the right psoas, the cross-sectional area (cm^2^) and CT attenuation were automatically calculated. The intramuscular adipose tissue area in the psoas was automatically calculated using the threshold between -190HU to -30HU. The FIR (%) was obtained by dividing the fatty area of the bilateral psoas by the total area of the bilateral psoas. PMI (bilateral psoas cross-sectional area adjusted to a patient’s height squared, cm^2^/m^2^), the mean bilateral CT attenuation and FIR were calculated. According to previous research carried out on Asian patients [10], a male PMI of < 5.923cm^2^/m^2^ or a female PMI of < 3.999 cm^2^/m^2^ was defined as sarcopenia.

### Statistical analysis

Quantitative data (such as CT attenuation, FIR, PMI, age, and BMI) were either represented as mean ± standard deviation, or as the median with an interquartile range. Qualitative data (such as gender, smoking or non-smoking, hypertension, and diabetes) were reported as counts and percentages. Statistical analyses were performed using SAS version 9.1 (SAS, Cary, NC, USA) and SPSS version 26.0 (SPSS Inc.). The intraclass correlation coefficient (ICC) was used to evaluate the repeatability of the psoas’ CT measurements. Quantitative variables were compared between the two groups by using an independent sample t-test or the Wilcoxon signed rank test. Qualitative variables were compared using the chi-square test. A logistic regression model was created to assess the potential influence of the psoas’ measurement parameters on having a risk of IH. The MedCalc software was used for receiver operating characteristic curve analysis to obtain the cut-off values for both the CT attenuation and FIR for IH. Statistical significance was defined at a *p* < 0.05 (2-sided).

## Results

One hundred twenty consecutive patients were included in this study. Their demographic and clinical characteristics are summarised in Table 1.

**Table 1:** Demographic and clinical characteristics of the included patients

Patient data	(*n* = 120)
Age (y)	62.5 ± 13.3
Gender (n, %)	
Male	55 (45.8%)
Female	65 (54.2%)
BMI^a^ (kg/m^2^)	25.9 (23.8–27.7)
< 18.5, underweight (n, %)	0 (0%)
18.5–25.0, normal weight (n, %)	49 (40.8%)
25.0–30.0, overweight (n, %)	58 (48.3%)
> 30, obesity (n, %)	12 (10%)
Smoking (n, %)	
Yes	84 (70.0%)
No	36 (30.0%)
Hypertension (n, %)	
Yes	58 (48.3%)
No	62 (51.7%)
Diabetes (n, %)	
Yes	23 (19.2%)
No	97 80.8%)

*BMI* Body mass index

^a^One patient’s BMI is missing as he was too weak to stand and have his weight measurement taken

### Repeatability of CT measurements

The results show that the inter-rater ICC of the psoas’ CT attenuation, muscle area, and FIR are 0.959, 0.996, and 0.914 (*P* < 0.001), respectively. It is suggested that the CT results’ repeatability is good, and that the analysis results in this article are credible.

### Comparison of demographic data and CT measurements between IH and non-IH groups (Table 2)

The psoas’ CT attenuation (t = − 2.18, *p* = 0.031) and PMI (Z=-2.03, *p* = 0.042) in the IH group were significantly lower than those in the non-IH group, and the FIR in IH patients was statistically higher than in non-IH patients (Z=3.57, *p* < 0.001). IH patients were, on average, 5 years older than non-IH patients (t = 2.26, *p* = 0.026). In addition, in the IH group, more than half were women (63.3%), while by contrast, females only accounted for 45.0% in the non-IH group (χ2 = 4.06, *p* = 0.044). The proportion of smokers in the IH group meanwhile was 1.4 times that of those in the non-IH group (χ2 = 7.78, *p* = 0.005). However, BMI, diabetes and hypertension were comparable between the two groups (*p* > 0.05).

**Table 2:** Comparison of demographic data and CT measurements between IH and non-IH groups

	IH (*n* = 60)	non-IH (*n* = 60)	Statistic	*P* value
CT attenuation (HU)	40.2 ± 7.6	43.0 ± 6.8	− 2.180	0.031^a^
FIR (%)	4.3 (3.1–5.5)	3.1 (2.0–4.2)	3.567	< 0.001^b^
PMI (cm^2^/m^2^)	5.0 (4.1–5.9)	5.9 ± 1.9	-2.034	0.042^b^
Age(y)	65.2 ± 11.9	59.8 ± 14.1	2.260	0.026^a^
Male (%)	36.70	55.00	4.062	0.044^c^
BMI^d^ (kg/m^2^)	26.1 (23.9–27.8)	25.6 (23.4–27.5)	-0.877	0.381^b^
Smoking (%)	81.70	58.30	7.778	0.005^c^
Hypertension (%)	50.00	46.67	0.134	0.715^c^
Diabetes (%)	18.30	20.00	0.054	0.817 ^c^

^a^independent sample t-test; ^b^Wilcoxon signed rank test; ^c^chi-square test

^d^One patient’s BMI is missing as he was too weak to stand and have his weight measurement taken

*IH* incisional hernias, *CT* computed tomography*, FIR* fatty infiltration rate, *PMI* psoas muscle index; *BMI* body mass index

### Logistic regression analysis of risk factors of IH

**Fig. 1:**
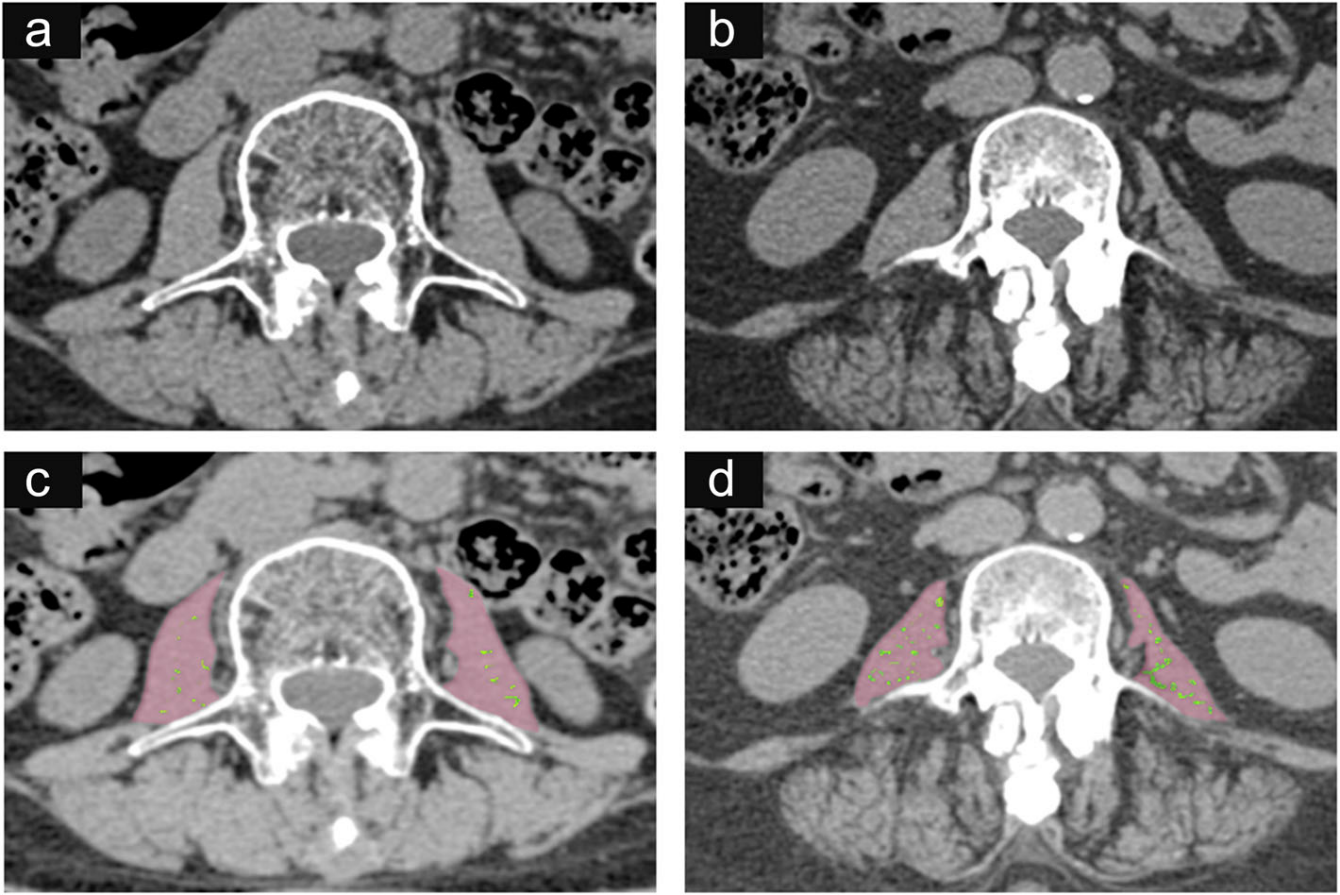
Measurement of the psoas. Images (**a** and **c**) are from a non-incisional hernia patient (64-year-old, female) and display a higher CT attenuation (47 HU) and a lower fatty infiltration rate (green, 2.1%); images (**b** and **d**) are from an incisional hernia patient (67-year-old, female) and exhibit a lower CT attenuation (24 HU) and a higher fatty infiltration rate (green, 9.4%). Their psoas muscle indexes are roughly equal (4.0 cm^2^/m^2^ vs. 4.1 cm^2^/m^2^)

The univariate logistic regression analysis (Table 3) shows that the psoas’ CT attenuation, FIR, PMI, age, gender and a history of smoking were all factors that were associated with IH. After adjusting for age, gender and a history of smoking in multivariate logistic regression analysis, CT attenuation was deemed an independent protective factor (OR 0.94, 95% CI 0.88-0.99, *p* = 0.042), and FIR an independent risk factor (OR 1.34, 95% CI 1.05-1.70, *p* = 0.018). By contrast, PMI (OR 0.78, 95% CI 0.56-1.07, *p* = 0.118) and sarcopenia (OR 0.84, 95% CI 0.38-1.87, *p* = 0.663) showed no effect on IH (Table 4) (Fig. 1).

**Table 3:** Univariate logistic regression analysis

	OR	95% CI	***P*** value
**CT attenuation**	0.95	0.90–0.99	0.034
**FIR**	1.35	1.10–1.66	0.004
**PMI**	0.76	0.60–0.96	0.021
**Sarcopenia**	0.80	0.37–1.71	0.559
**Age**	1.03	1.00–1.06	0.028
**Gender**	2.11	1.02–4.39	0.045
**BMI**	1.00	0.91–1.10	0.972
**Smoking**	3.18	1.39–7.30	0.006
**Hypertension**	1.14	0.56–2.34	0.715
**Diabetes**	0.90	0.36–2.23	0.817

*CT* Computed tomography, *FIR* Fatty infiltration rate, *PMI* Psoas muscle index, *BMI* Body mass index

**Table 4:** Multivariate logistic regression analysis

		OR	95% CI	***P*** value
**CT attenuation**		0.94	0.88–0.99	0.042
	**Age**	0.75	0.24–2.32	0.614
	**Gender**	0.96	0.38–2.45	0.928
	**Smoking**	3.31	1.19–9.22	0.022
**FIR**		1.34	1.05–1.70	0.018
	**Age**	0.77	0.25–2.34	0.647
	**Gender**	0.85	0.32–2.21	0.732
	**Smoking**	2.75	1.00–7.53	0.049
**PMI**		0.78	0.56–1.07	0.118
	**Age**	1.07	0.38–2.98	0.897
	**Gender**	0.71	0.23–2.22	0.556
	**Smoking**	3.06	1.11–8.44	0.031
**Sarcopenia**		0.84	0.38–1.87	0.663
	**Age**	1.29	0.47–3.56	0.621
	**Gender**	1.26	0.52–3.07	0.612
	**Smoking**	2.73	1.01–7.39	0.047

*CT* Computed tomography, *FIR* Fatty infiltration rate, *PMI* Psoas muscle index

**Fig. 2:**
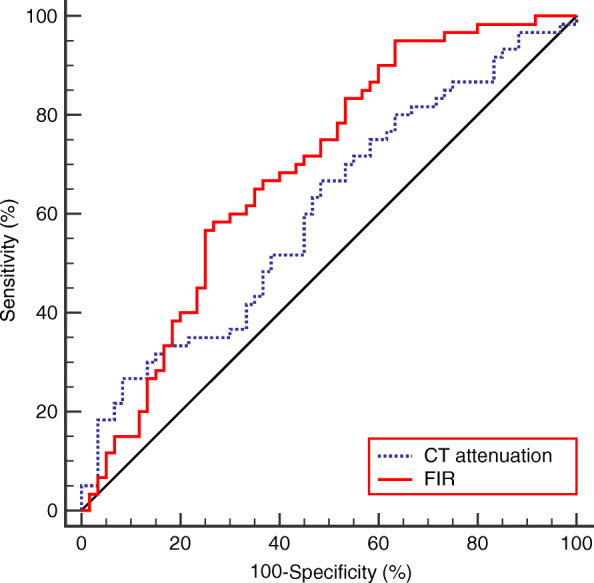
The receiver operating characteristic curves for CT attenuation and fatty infiltration rate. The fatty infiltration rate has a higher diagnostic value than CT attenuation. The area under the curve of the fatty infiltration rate for diagnosing incisional hernia is 0.689 (95% CI 0.60–0.77), with a cut-off at 3.8%, sensitivity of 58.33%, and specificity of 73.3%. The area under the curve of the CT attenuation for diagnosing incisional hernia is 0.604 (95% CI 0.51–0.69), with a cut-off at 43.5 HU, sensitivity of 66.67% and specificity of 51.67%

As shown in Fig. 2, the area under the curves of the CT attenuation and FIR are 0.604 (95% CI 0.51-0.69), and 0.689 (95% CI 0.60-0.77), respectively. FIR had a significantly higher diagnostic efficacy than the CT attenuation (Z = 2.610, *p* = 0.009). The cut-off value for FIR was 3.8%, with a sensitivity of 58.33% and a specificity of 73.33%. The cut-off value for CT attenuation, was 43.5 HU, with a sensitivity of 66.67% and a specificity of 51.67%.

## Discussion

In this cross-sectional study, we recruited patients who had undergone an appendicectomy due to appendicitis, and we divided them into two groups based on whether they had developed an IH or not. We explored the relationship between the psoas’ CT measurements and IH. After adjusting for age, gender and whether the participants were smoking or non-smoking, the psoas’ CT attenuation was deemed a protective factor for IH, and FIR was deemed a risk factor. In addition, it was also noted that PMI and sarcopenia hardly had an effect on the occurrence of IH at all.Psoas atrophy is mainly manifested by volume reduction and morphological changes, with an unobvious deposition of fat. Therefore, previous studies on IH have mainly focused on the psoas’ muscle area and skeletal muscle index. However, in addition to volume reduction, muscle atrophy may pathologically exhibit a fatty infiltration and muscle fibre loss [3, 11]. Some authors believe that CT attenuation may accurately reflect the number of muscle fibres and the degree of fat deposition [12]. Muscle CT attenuation is related to various diseases: paraspinal muscle density is associated with facet joint osteoarthritis, spondylolisthesis and disc narrowing at the same level [13]. Lower thigh muscle CT attenuation could increase the risk of hip fracture, and a standard deviation decrease of just 1 in the thigh muscle HU value conferred a nearly 40% increase in the risk of hip fracture [14]. For critically ill adult patients being treated with mechanical ventilation systems, those with a lower skeletal muscle CT value at admission had a higher 6-month mortality rate, and a 10 HU increase in muscle density was associated with a 14% decrease in hospital lengths of stay [15]. Furthermore, cancer patients with cachexia and low muscle CT values had a poor prognosis [16].

Skeletal muscle density inversely correlated with the length of hospitalisation when following complex abdominal wall hernia surgery [17]. In our study, patients with a lower psoas CT attenuation were more likely to develop IH. It could be that the CT attenuation of the psoas correlates with that of the abdominal wall muscles [4]. A decreased CT attenuation of the muscles is independently associated with muscle weakness [2]. Therefore, a lower CT attenuation of the psoas indirectly reflects a weakness of the abdominal wall muscles, which are susceptible to hernias [18].

In our study, patients with a higher FIR of the psoas were prone to develop IH. Fatty infiltration in skeletal muscle has been identified as a possible cause for loss of muscle quality [2]. Fatty infiltration induces insulin resistance, which impairs the normal capacities for protein synthesis, and subsequently contributes to muscle atrophy [19, 20]. In previous studies, MRI has often been applied to quantitatively measure the muscle’s fat content [21, 22], and they concluded that paraspinal fatty infiltration, rather than the muscle’s cross-sectional area, was associated with high-intensity pain/disability and structural abnormalities in the lumbar spine [23]. Moreover, in patients with L4–5 single-segment degenerative lumbar spinal stenosis, a fatty infiltration in the multifidus muscles at L5-S1 could be correlated with the disc bulge at the stenosis segment and the reduction of lumbar lordosis [24]. Since MRI examinations are not routinely performed on hernia patients, the MRI-measured muscle fat content in these patients is not widely taken into consideration. Previous published research on CT imaging mostly used HU value to indirectly represent muscle fat content. However, since muscle CT attenuation can be affected by previous surgeries and the deposition of high-density substances, such as calcium and bleeding, it may not effectively reflect the muscle’s fat content. Wang C et al. reported that the psoas’ CT attenuation in patients with osteoporosis fractures was unexpectedly higher than those without osteoporosis fractures [25], which may be related to intra-muscular haemorrhages or muscle repair following a fracture. In this case, CT attenuation cannot accurately reflect the muscle’s fibre content or the degree of fat accumulation. Although relatively complex, measuring the intramuscular fat area or muscle FIR by defining a CT threshold can more accurately reflect the degree of muscle fatty infiltration. So far, few studies have used CT to evaluate fatty infiltration in the muscle. One study that did, by Peter et al., showed that as shoulder strength increases following a shoulder arthroplasty, the rotator cuff fatty infiltration, measured by CT, decreases [26]. To our knowledge, this is the first study to explore the potential relationship between the FIR of the psoas and IH. We concluded that when compared with CT attenuation, FIR was more closely related to IH.

Previous studies that have investigated the roles of abdominal muscles in malignancies, mostly measured the muscle area in the L3 or L4 cross-sectional image, including the psoas, erector spinae, quadratus lumborum, transversus abdominus, rectus abdominus, and the internal and external obliques. The corresponding skeletal muscle index might predict the prognosis of various malignancies [9, 27]. CT-determined sarcopenia, that was determined by measuring the level L3 cross-section muscles area, was not a risk factor for the occurrence of IH [28]; although it could prolong the postoperative hospital stay [17]. It is very labour-intensive to measure all the muscle areas in a cross-sectional image, since it must be performed using a specific post-processing software by defining the range of the CT values to exclude intermuscular fat. To only measure the psoas area at level L3 is simple however, and the corresponding muscle index (PMI) correlates with the whole-body muscle mass [29]. A decrease in PMI indicates a decline in the whole-body muscle mass (including the abdominal wall muscles), which results in a decreased functional capacity. Thus, having a low PMI may constitute a potential risk factor for IH. Although PMI at level L3 was a protective factor for IH in our research’s univariate regression analysis, it was not statistically significant in multivariate regression analysis. Sarcopenia meanwhile, as defined by PMI’s cut-off values in a previous study (based on an Asian population), was not associated with IH in neither the univariate nor multivariate regression analyses.

Our study demonstrated that psoas’ CT attenuation and FIR were associated with IH, but PMI and sarcopenia were not. PMI and sarcopenia reflect muscle mass, while CT attenuation and FIR are associated with psoas’ intramuscular adipose. We therefore speculate that the occurrence of IH is more related to muscle quality than quantity.

Among our study population, smoking was deemed a significant risk factor for IH, and this is consistent with previous publications [30, 31]. One hypothesis is that tobacco leads to atheroma, which reduces the blood supply to the abdominal wall. In addition, a decreased collagen deposition in surgical test wounds has been found among smokers [32].

In our study, BMI was not deemed an influencing factor of IH. However, many studies have shown that BMI, especially BMI > 30 (obesity), is a risk factor for IH [33, 34]. Obesity is likely to increase intra-abdominal pressure by putting mechanical stress on the abdominal incision, thus predisposing the occurrence of a hernia. However, the BMI in our population (26.1 in the IH group, 25.6 in the non-IH group) is lower than in those studies, and only 10.0% of our patients had a BMI > 30. Therefore, BMI may not have identified as a risk factor because the majority of our patients were non-obese.

Our univariate analysis identified older age and female sex as significant risk factors for IH, while no significant difference was noted in the multivariate analysis. Different studies report inconsistent results on whether age or gender are risk factors for incisional hernias [35–37]. These discrepancies in the age-specific or sex-specific influences on the risk for IH may reflect differences in the study population and diseases. We also showed two non-significant trends in the multivariate analysis: hypertension and diabetes, both of which are consistent with previous research [35, 36].

This study has several limitations. First, wound closure techniques and wound infection are recognised risk factors for IH. However, some patients could not recall the relevant information, since more than 10 years or even decades had passed since their appendectomy to this admission. Taking recall-bias into consideration, wound closure techniques and wound infection were not included in the statistical analysis. Second, the area under the curves of CT attenuation and FIR were not very high in our study. The purpose of our study was to screen the influencing factors of IH after appendectomy, and to provide a basis for further research. A perfect IH prediction model will be constructed in our follow-up research. Third, the cross-sectional retrospective study design limits our ability to ascertain causality and may cause the inevitability of selection bias. Thus, our conclusion needs to be verified using a larger sample size.

## Conclusions

In summary, our study indicates that following an appendectomy, a decreased CT attenuation and increased FIR of the psoas are risk factors for IH. For patients who have had an appendectomy, when there is a low psoas CT attenuation or high psoas FIR, a prophylaxis of the hernia should be conducted to reduce the possibility of the occurrence of IH.

## Data Availability

The data sets generated during and analysed during the current study are not available due to hospital regulation, but are available from the corresponding author on reasonable request.

## Abbreviations

BMI: Body mass index
CT: Computed tomography
FIR: Fatty infiltration rate
HU: Hounsfield unit
IH: Incisional hernias
MRI: Magnetic resonance imaging
PMI: Psoas muscle index
